# Clinicopathological features of different subtypes in adenomyosis: Focus on early lesions

**DOI:** 10.1371/journal.pone.0254147

**Published:** 2021-07-14

**Authors:** Hiroshi Kobayashi, Sho Matsubara, Shogo Imanaka

**Affiliations:** 1 Department of Obstetrics and Gynecology, Nara Medical University, Kashihara, Nara, Japan; 2 Ms. Clinic MayOne, Kashihara, Nara, Japan; Dipartimento di Scienze Mediche e Chirugiche (DIMEC), Orsola Hospital, ITALY

## Abstract

**Background:**

The aim of this study is to investigate the clinicopathological features of intrinsic and extrinsic subtypes in adenomyosis. In particular, we focused on the early lesions of adenomyosis.

**Methods:**

This is a single-center, prospective study of women who elected surgery for adenomyosis at the Department of Gynecology, Nara Medical University Hospital, Kashihara, Japan, from April 2008 to March 2018. Adenomyosis was histologically classified as intrinsic, extrinsic, and others, depending on the type of intramural growth. Adenomyosis that occurs at the inner and outer myometrium was defined as an intrinsic and extrinsic type, respectively.

**Results:**

One hundred eighty-nine patients with histologically confirmed adenomyosis were classified into three different types, 74 intrinsic type, 78 extrinsic type, and 37 other type. Compared to the intrinsic type, the extrinsic type was more likely to have endometriosis, including ovarian endometrioma (OMA), superficial peritoneal endometriosis (SUP), or deep infiltrating endometriosis (DIE). To further identify the clinicopathological features of early-stage adenomyosis, we focused only on patients with intrinsic and extrinsic types of adenomyosis with less than one-third of muscular layer infiltration. Patients with early-stage intrinsic adenomyosis were more likely to experience induced abortions. Patients with early-stage extrinsic adenomyosis were more likely to have endometriosis. The coexistence of endometriosis and the lack of induced abortion were independent predictors of extrinsic adenomyosis. Multivariate logistic regression analysis identified coexistence of endometriosis as independent predictors of the early stage extrinsic adenomyosis.

**Conclusion:**

The study suggests that there are at least two types of adenomyosis, where the intrinsic type is closely associated with a history of induced abortion, while the extrinsic type is strongly associated with endometriosis. Adenomyosis might be a gynecological disorder with complex pathogenesis implicating both traumatic and endometriotic factors.

## Introduction

Adenomyosis is a common uterine disorder characterized by the presence of endometrial glands and stromas within the myometrium [1]. Symptoms are non-specific and are related to an enlarged uterus, menorrhagia, abnormal uterine bleeding, pelvic pain, or infertility; a third of patients can be asymptomatic [2–5]. Effective management of adenomyosis requires a lifelong plan as the disease has a negative impact on quality of life in terms of menstrual symptoms, fertility, and has a high risk of miscarriage, obstetric complications and poor pregnancy outcomes [1]. Furthermore, adenomyosis often coexists with other gynecological conditions, such as endometriosis and uterine fibroids, that share several symptoms [1] and its treatment is still challenging [3]. Traditionally, adenomyosis has been a histological diagnosis at hysterectomy [3]. Adenomyosis has always been considered the classic condition discovered in multiparous women over 40 years old who have menorrhagia and dysmenorrhea [1,6]. Nowadays, advances in modern imaging technology, including transvaginal ultrasound and magnetic resonance imaging (MRI), have made it possible to diagnose different phenotypes (diffuse or localized) of adenomyosis [7]. The detection of the "question mark" sign by transvaginal ultrasound is effective for the diagnosis of adenomyosis [8]. In young girls in their 10s to 20s with a history of chronic pelvic pain, the prevalence of adenomyosis reached 46.0% [9]. Diffuse adenomyosis may also develop in younger nulligravid women (early 20 years) than previously thought [7]. Thus, adenomyosis is not a disease of the elderly.

To date there is no unified classification system, but several image-based classifications of adenomyosis have been proposed [10–14]. The history of the classification of adenomyosis is summarized in ref. [11]. Just 100 years ago, Sampson divided adenomyosis into several groups according to the origin or pathogenesis [3,15]. This theory, in turn, led to Kishi’s classification criteria in 2012 [10]. The authors divided adenomyosis into at least three groups: adenomyosis resulting from invagination of the endometrial basalis into the myometrium; adenomyosis caused by endometriosis infiltration from outside the uterus; and adenomyosis possibly arising from Müllerian remnants [10]. There are many other types of adenomyosis, including intrinsic adenomyosis, extrinsic adenomyosis, adenomyosis externa and focal adenomyosis located in the outer myometrium (FAOM) [7,11,16]. The two types of adenomyosis, intrinsic and extrinsic, can be more clearly distinguished histopathologically. Intrinsic and extrinsic adenomyosis occur in the inner and outer layers of the uterus, respectively. It has been suggested that these two types of adenomyosis have different clinicopathological characteristics and pathogenesis [10]. However, it is unclear whether there are already differences in clinicopathological features in early-stage adenomyosis. Long-term prospective cohort studies of asymptomatic young women are needed to detect early lesions of adenomyosis, but such clinical trials are actually difficult. Therefore, we classified histologically diagnosed adenomyosis into intrinsic, extrinsic and other types, and further focused on the early lesions of intrinsic and extrinsic adenomyosis. The purpose of this study is to identify the clinicopathological features associated with intrinsic and extrinsic adenomyosis, especially in early-stage patients.

## Materials and methods

### Patient selection and analytic cohort

A single-center prospective cohort (DoG-NaMe) study was conducted by collecting data from patients admitted to the Department of Gynecology, Nara Medical University Hospital, Kashihara, Japan. The DoG-NaMe study consists of an endometriosis cohort, an adenomyosis cohort, and an ovarian cancer cohort. We performed an observational cross-sectional study using data from the adenomyosis cohort study from April 2008 to March 2018. We used data from patients who met all three selection criteria: 1) patients undergoing surgery with removal of lesions for histological evaluation; 2) patients with pathological confirmation of adenomyosis; and 3) patients who underwent magnetic resonance imaging (MRI) examinations prior to surgery. The criteria for exclusion were: 1) age below 20 years; 2) active surveillance only; 3) women coexisting with malignancies; and 4) incomplete data. Patients with preoperative use of hormone therapy were not excluded from the study. Many patients with suspected adenomyosis on transvaginal ultrasonography were referred to this university hospital for surgery from a nearby clinic. These patients were recruited for this study. MRI is mandated by this protocol as a requirement for surgery or surveillance. MRI protocol included T1w and T2w sequences using a 3T system (Magnetom Verio, Siemens Healthcare, Erlangen, Germany). MRI and blood tests were performed within a month of surgery. Information on demographic data, medical history, and clinicopathological characteristics was collected from a database containing comprehensive medical records and pathology reports. Patients’ medical records were anonymous. Clinicopathological variables include age at surgery, gravidity, parity, number of caesarean sections, number of induced abortion, BMI, severity of symptoms such as pelvic pain, menorrhagia, and infertility, coexistence of ovarian endometrioma (OMA), deep infiltrating endometriosis (DIE) and/or superficial peritoneal endometriosis (SUP), coexistence of submucosal, intramural, or sub-serosal fibroids, maximum length from cervix to uterine fundus, the length of the thickest wall [either], the length of the thickest wall [sum], the length of the thickest lesion, anteflexed or midline/retroflexed, hemoglobin levels, CA125 levels, and preoperative hormone therapy with combined oral contraceptives (low-dose estrogen-progestin combinations), dienogest, or gonadotropin-releasing hormone (GnRH) agonists. We investigated the prevalence of OMA, SUP, DIE, and uterine fibroids. Ethical approval was obtained from the Nara Medical University Ethics Committee (2012–541) and informed written consent was obtained from all participants.

### Classification based on the affected area and extent of adenomyosis

We used a simplified classification system based on the affected area and the locoregional extension of adenomyosis lesions [11]. Patients with adenomyosis were categorized into intrinsic, extrinsic, and other types. The intrinsic type (denoted as group A) is defined as adenomyosis that occurs in the uterine inner layer without affecting the outer structures of the myometrium. The extrinsic type (denoted as group B) is defined as adenomyosis that occurs in the uterine outer layer without affecting the inner structures. The extent of adenomyosis lesion is further categorized into three volumes (<1/3, <2/3, or >2/3 of uterine wall). A1, A2 and A3 are defined as "the lesion is confined to the inner 1/3 of the uterine myometrium", "the lesion is confined to the inner 2/3 of the uterine myometrium", and "the lesion extends beyond the inner 2/3 of the myometrium and part of the lesion reaches the uterine serosa." B1, B2, and B3 are defined as "the lesion is confined to the outer 1/3 of the uterine myometrium", "the lesion is confined to the inner 2/3 of the uterine myometrium", and "the lesion extends beyond the outer 2/3 of the myometrium and part of the lesion reaches the uterine endometrium." If the lesion extends to the entire myometrium, A3 and B3 are indistinguishable by MRI and pathology. If either of the two gynecologists diagnosed the patient as neither type A nor type B, she was classified as "unclassifiable". Patients were classified as "other type" when the two gynecologists agreed that they did not belong to either type A or type B. In this paper, we adopted the classification of "other type". When adenomyosis coexists with uterine fibroids, it is necessary to define the type and extension of adenomyosis. For example, in patients with intrinsic adenomyosis, if the adenomyosis lesion is 30 mm in thickness and a fibroid 70 mm in diameter is localized outside the lesion, this case was classified as "A1". The thickest wall [either] was measured at the thickest length of either the anterior or posterior wall of the uterus. The thickest wall [sum] was measured at the thickest length of the sum of the anterior and posterior walls of the uterus. The thickest lesion was measured at the thickest adenomyosis lesion infiltrating the uterine myometrium.

### Definition of pelvic pain, menorrhagia, and infertility

Pelvic pain is defined as pain that required the use of painkillers and affected daily life (the loss or reduction of daily activities). Visual Analog Scale (VAS) scores can be evaluated in four levels: painless (score, 0), mild (score, 1–3), moderate (score, 4–7), and severe (score, 8–10). Patients with a score of 8–10 points were determined to have pelvic pain. Heavy menstrual bleeding was defined when one or more of the following are true: use one or more sanitary napkins or tampons within one hour over several hours; use of dual sanitary protective equipment to control menstruation; awake at midnight to change sanitary protection; bleeding lasting more than 1 week; excretion of blood coagulation over one quarter of the napkin area; and limitation of daily activities due to anemia symptoms such as severe menstruation, fatigue, shortness of breath. This is an excerpt from the Mayo Clinic homepage [17]. There were 2 types of patients in the infertility group: Patients who failed to achieve a clinical pregnancy following ≥12 months of regular unprotected sexual intercourse [18] and those who have already been treated at fertility hospitals.

### Quantification of serum CA125

Blood samples were obtained from all study participants to determine serum CA125 levels at least 4 weeks prior to surgery. The blood samples were centrifuged at 1500×g for 10 minutes at 4°C, and the serum was stored at −20°C until used for measurements. Serum CA125 concentrations were determined using an electrochemiluminescence Elecsys immunoassay (ECLIA) (Roche Diagnostics, Salzburg, Austria).

### Statistical analysis

Statistical analyses were performed using SPSS Statistics version 25 (IBM Japan). The data were presented as mean and standard deviation (SD) or median and range. Data distribution was verified by the Shapiro-Wilk test. t-test was conducted for mean comparison for the groups. Pearson’s chi-squared test (χ2) was applied to categorize variables. Data were analyzed using the Kruskal-Wallis test followed by post hoc analysis with Bonferroni correction to evaluate differences among the 3 groups. The nonparametric Mann-Whitney U-test was used for statistical analysis of the findings, due to the abnormal distribution of the data obtained for A1 and B1 groups. Multivariate logistic regression analysis was performed on the significant factors (P<0.05) from univariate analysis using a Cox proportional hazards model to identify independent predictors of extrinsic adenomyosis.

P-values of <0.05 were considered to indicate statistically significant differences.

## Results

### Clinicopathological variables in patients with intrinsic, extrinsic and other types of adenomyosis

During the period of this study, 230 patients were diagnosed with adenomyosis by MRI. Of the 230, 197 underwent surgery. Of these, 189 patients were histologically confirmed to have adenomyosis. This study revealed that the diagnostic sensitivity of adenomyosis by MRI was as high as 95.9%. The remaining 33 women were under active surveillance for adenomyosis management and were not included in this study. This study examined the clinicopathological features of 189 patients with histologically confirmed adenomyosis. This cohort of patients included 74 women with intrinsic adenomyosis (20 for A1 and 54 for A2), 78 extrinsic adenomyosis (43 for B1 and 35 for B2), and 37 other types of adenomyosis. First, clinicopathological variables were compared in the three groups. Clinicopathological characteristics of the three groups are presented in Table 1. Of the 60 patients with adenomyosis coexisting with DIE and/or SUP, 7 had DIE only, 3 had SUP only, and 50 had both DIE and SUP. Therefore, the clinicopathological variables were grouped as "coexistence of DIE and/or SUP". Deep endometriosis infiltrated rectum and uterosacral ligaments (n = 30, 52.6%), rectum and sigmoid colon (n = 19, 33.3%), only the sigmoid colon (n = 3, 5.3%), and rarely ureters (n = 6, 10.5%), bladder (n = 5, 8.7%), small bowel (n = 2, 3.5%), cecum (n = 1, 1.7%), and others (n = 5, 8.8%). The following five variables, including coexistence of OMA (P < 0.001), coexistence of DIE and/or SUP (<0.001), the thickest wall [either] (<0.001), the thickest wall [sum] (0.039) and the thickest lesion (<0.001), were significantly different among the 3 groups (by Kruskal-Wallis test). The prevalence of OMA was significantly higher in the extrinsic group (50/78, 64.1%) compared to the intrinsic (4/74, 5.4%) and other (9/37, 24.3%) groups (P < 0.001 by Kruskal-Wallis test and post hoc analysis with Bonferroni correction). The prevalence of DIE and/or SUP was also highest in the extrinsic group (50/78, 64.1%) among the three groups. Not surprisingly, among the three groups, uterine myometrium and adenomyosis lesions were the thickest in the other group (by Kruskal-Wallis test), but there was no significant difference between the intrinsic and extrinsic groups (by Kruskal-Wallis test and post hoc analysis with Bonferroni correction). For the other variables, there were no statistical differences between the three groups (P> 0.05 by Kruskal-Wallis test).

**Table 1:** Clinicopathological variables in patients with intrinsic, extrinsic and other types of adenomyosis.

Variables	Intrinsic adenomyosis n = 74	Extrinsic adenomyosis n = 78	Unclassified n = 37	p
Age at surgery				
Median (Range)	44(33–55)	43(21–52)	43(29–52)	0.981
Mean ± SD	44.3 ± 5.3	42.5 ± 6.1	41.9 ± 5.1	
Gravidity				0.048
0	19(25.7)	27(34.6)	8(21.6)	
1	10(13.5)	16(20.5)	12(32.4)	
>1	45(60.8)	35(44.9)	17(45.9)	
Parity				0.312
0	18(24.3)	32(41.0)	12(32.4)	
1	13(17.6)	21(26.9)	13(35.1)	
>1	43(58.1)	25(32,1)	12(32.4)	
Number of caesarean sections				0.144
0	67(90.5)	65(83.3)	33(89.2)	
1	4(5.4)	9(11.5)	4(10.8)	
>1	3(4.1)	4(51.3)	0(0.0)	
Number of induced abortion				0.085
0	39(52.7)	68(87.2)	28(75.7)	
1	19(25.7)	7(9.0)	7(18.9)	
>1	16(21.6)	3(3.8)	2(5.4)	
BMI	22.5(13.0–39.0)	21.2(16.0–36.0)	22.5(18.0–31.0)	0.548
Pelvic pain				0.984
No	20(27.0)	21(26.9)	10(27.0)	
Yes	54(73.0)	57(73.1)	27(73.0)	
Menorrhagia				0.502
No	26(35.1)	33(42.3)	14(37.8)	
Yes	48(64.9)	45(57.7)	23(62.2)	
Infertility				0.676
No	71(95.9)	53(67.9)	14(37.8)	
Yes	3(4.1)	25(32.1)	23(62.2)	
Coexistence of OMA				<0.001
No	70(94.6)	28(35.9)	28(75.7)	
Yes	4^a^(5.4)	50^b^(64.1)	9^c^(24.3)	
Coexistence of DIE and/or SUP				<0.001
No	69(93.2)	28(35.9)	32(86.5)	
Yes	5^d^(6.8)	50^e^(64.1)	5^f^(13.5)	
Coexistence of intramural or sub-serosal fibroids				0.178
No	36(48.6)	35(44.9)	22(59.5)	
Yes	38(51.4)	43(55.1)	15(40.5)	
Coexistence of submucosal fibroids				0.362
No	57(77.0)	64(82.1)	34(91.9)	
Yes	17(23.0)	14(17.9)	3(8.1)	
Maximum length from cervix to uterine fundus, mm	105.0(50.0–212.0)	100.5(61.0–165.0)	107.0(76.0–182.0)	0.072
The length of the thickest wall [either], mm	38.0 ^g^(14.0–76.0)	31.5 ^h^(11.0–78.0)	44.5 ^i^(8.0–85.0)	<0.001
The length of the thickest wall [sum], mm	58.5 ^j^(15.0–116.0)	52.5 ^k^(21.0–112.0)	64.5 ^l^(11.0–144.0)	0.039
The length of the thickest lesion, mm	26.0 ^m^(2.0–62.0)	21.5 ^n^(4.0–68.0)	42.0 ^o^(12.0–84.0)	<0.001
Anteflexed and midline/retroflexed				0.745
Anteflexed	56(75.7)	46(59.0)	26(70.3)	
Midline and retroflexed	18(24.3)	32(41.0)	11(29.7)	
Hemoglobin, mg/dl	12.3(7.3–16.0)	11.7(4.6–15.3)	11.8(8.5–13.9)	0.495
CA125, U/ml	30.0(6.0–1706.0)	38.0(1.0–659.0)	51.5(9.0–268.0)	0.715
Combined oral contraceptives				0.831
No	64(86.5)	62(79.5)	29(78.4)	
Yes	10(13.5)	16(20.5)	8(21.6)	
Dienogest				0.253
No	59(79.7)	56(71.8)	21(56.8)	
Yes	15(20.3)	22(28.2)	16(43.2)	
GnRH agonists				0.051
No	60(81.1)	66(84.6)	25(67.6)	
Yes	14(18.9)	12(15.4)	12(32.4)	

Except for the Range, the numbers in parentheses indicate percentages.

a vs. b, P <0.001; b vs. c, P <0.001; a vs. c, P = 0.003.

d vs. e, P <0.001; e vs. f, P <0.001; d vs. f, P = 0.153.

g vs. h, P = 0.475; h vs i, P = 0.008; g vs. i, P = 0.025.

j vs. k, P = 0.076; k vs. l, P = 0.031; j vs. l, P = 0.626.

m vs. n, P = 0.209; n vs. o, P <0.000; m vs. o, P <0.001.

In addition, clinicopathological variables were analyzed in patients with early-stage adenomyosis, groups A1 and B1. The number of induced abortion (P = 0.017), the prevalence of OMA (P <0.001) and DIE and/or SUP (P <0.001) were significantly different between the two groups (Table 2). The A1 group has experienced significantly more induced abortions than the B1 group (45.0% vs. 14.0%, P = 0.017). Compared to the A1 group, the B1 group coexisted significantly more with OMA (51.2% vs. 5.0%, P <0.001) and DIE and/or SUP (51.2% vs. 0%, P <0.001). There were no statistical differences in the other variables between the two groups (P> 0.05).

**Table 2:** Clinicopathological variables in patients with A1 and B1 adenomyosis.

Variables	A1 n = 20	B1 n = 43	p
Age at surgery			
Median, Range	44(33–50)	45(21–52)	0.960
Mean ± SD	43.500 ± 4.662	43.419 ± 6.558	
Gravidity			0.070
0	4(20.0)	13(30.2)	
1	3(15.0)	9(20.9)	
>1	13(65.0)	21(48.8)	
Parity			0.666
0	7(35.0)	16(37.2)	
1	2(10.0)	10(23.3)	
>1	11(55.0)	17(39.5)	
Number of caesarean sections			0.727
0	18(90.0)	36(83.7)	
1	0(0.0)	4(9.3)	
>1	2(10.0)	3(7.0)	
Number of induced abortion			0.017
0	11(55.0)	37(86.0)	
1	6(30.0)	4(9.3)	
>1	3(15.0)	2(4.7)	
BMI	22.0(17.0–36.0)	21.0(16.0–36.0)	0.420
Pelvic pain			0.450
No	9(45.0)	15(34.9)	
Yes	11(55.0)	28(65.1)	
Menorrhagia			0.417
No	12(60.0)	21(48.8)	
Yes	8(40.0)	22(51.1)	
Infertility			0.178
No	20(100.0)	31(72.1)	
Yes	0(0.0)	12(27.9)	
Coexistence of OMA			<0.001
No	19(95.0)	21(48.8)	
Yes	1(5.0)	22(51.1)	
Coexistence of DIE and/or SUP			<0.001
No	20(100.0)	21(48.8)	
Yes	0(0.0)	22(51.1)	
Coexistence of intramural or sub-serosal fibroids			0.818
No	9(45.0)	18(41.9)	
Yes	11(55.0)	25(58.1)	
Coexistence of submucosal fibroids			0.882
No	15(75.0)	33(76.7)	
Yes	5(25.0)	10(23.3)	
Maximum length from cervix to uterine fundus, mm	89.0(69.0–145.0)	90.0(61.0–148.0)	0.680
The length of the thickest wall [either], mm	27.0(14.0–58.0)	25.0(11.0–78.0)	0.843
The length of the thickest wall [sum], mm	48.5(30.0–90.0)	42.0(21.0–112.0)	0.258
The length of the thickest lesion, mm	16.0(2.0–36.0)	12.5(4.0–36.0)	0.362
Anteflexed and midline/retroflexed			0.472
Anteflexed	14(70.0)	26(60.5)	
Midline and retroflexed	6(30.0)	17(39.5)	
Hemoglobin, mg/dl	12.7(7.6–15.1)	11.4(4.6–15.3)	0.217
CA125, U/ml	21.0(6.0–915.0)	31.0(1.0–125.0)	0.215
Combined oral contraceptives			0.899
No	17(85.0)	36(83.7)	
Yes	3(15.0)	7(16.3)	
Dienogest			0.300
No	19(95.0)	37(86.0)	
Yes	1(5.0)	6(14.0)	
GnRH agonists			0.320
No	17(85.0)	30(69.8)	
Yes	3(15.0)	13(30.2)	

Except for the Range, the numbers in parentheses indicate percentages.

Univariate analysis identified three variables as predictors of the early stage extrinsic adenomyosis: coexistence of DIE and/or SUP, coexistence of OMA, and no previous history of induced abortion (P <0.001, <0.001, and 0.017) (Table 3). On multivariable analysis, coexistence of DIE and/or SUP and coexistence of OMA were independent predictors.

**Table 3:** Univariate and multivariate analysis to identify independent predictors of the early stages of extrinsic adenomyosis.

Variable	Univariate analysis P value	Multivariate analysis	
		HR (95% CI)	P value
Coexistence of DIE and/or SUP	<0.001	9.546 (2.570–31.841)	<0.001
Coexistence of OMA	<0.001	5.021 (1.523–14.027)	0.004
Number of induced abortion	0.017	1.337 (0.897–3.275)	0.078

## Discussion

The present study identified phenotype-specific risk factors with a particular focus on patients with early lesions of intrinsic and extrinsic adenomyosis. Based on surgical pathological findings, patients with adenomyosis were classified into intrinsic (A1 and A2), extrinsic (B1 and B2), and other types (A3 and B3). This study revealed that 1) patients with extrinsic adenomyosis were more likely to present with endometriosis than the other two groups; 2) similar results were obtained even when limited to patients with early lesions of intrinsic and extrinsic adenomyosis (the A1 and B1 groups); and 3) the A1 group was more likely to present with a history of induced abortion than the B1 group.

First, our study showed that patients with extrinsic adenomyosis were much more likely to present with OMA, SUP, or DIE compared to those with intrinsic adenomyosis. Adenomyosis often coexists with endometriosis and uterine fibroids [5,19,20]. A retrospective population-based cohort study demonstrated that associated symptoms (menorrhagia or abnormal uterine bleeding, dysmenorrhea or pelvic pain, dyspareunia, and infertility) were observed in 90.8%; 18.0% had co-occurrent endometriosis and 47.6% had co-occurrent uterine fibroids [21]. This cohort study did not reveal the prevalence of concurrent endometriosis and associated symptoms in different phenotypes of adenomyosis. The fact that the B1 group was more likely to present with endometriosis than the A1 group suggests that early lesions in patients with extrinsic adenomyosis may be associated with endometriosis. In fact, endometriosis coexisted in 51.2% (22/43) of the B1 group and 80.0% (28/35) of the B2 group, respectively. In addition, the prevalence of endometriosis in the A1 and A2 groups was as low as 5% (1/20) and 9% (5/54), respectively, suggesting that intrinsic adenomyosis is less associated with endometriosis. Clinicians recognize that some adenomyosis did not affect the inner layer of the myometrium, but occurred in the outer shell of the uterus [9,22]. This type can be categorized into extrinsic adenomyosis. Our data also support the previous results that posterior cul-de-sac endometriosis, adhesion and posterior wall involvement are quite frequent in extrinsic adenomyosis that appears to be a result of the direct invasion of ectopic endometrial tissues [9,22]. Assuming that intrinsic and extrinsic adenomyosis occur by invagination of the basalis endometrium into the myometrium and by direct infiltration from endometriosis, respectively, the two types of adenomyosis are thought to originate from the basal layer and the functional layer of the eutopic endometrium. There was also no significant difference in the frequency of coexistence of uterine fibroids between the three groups, and adenomyosis appears to occur independently of uterine fibroids.

Three papers related to our study have recently been published. Li et al. presented clinical and pathological features of adenomyosis with or without coexisting endometriosis [23]. Two hundred and eight patients were surgically treated, but only 83 underwent hysterectomy. Multivariate logistic regression analysis revealed that patients in the EM group had an earlier age of menarche (P = 0.036), more frequent rectal irritation symptoms (P = 0.038), and smaller uterine volume (P = 0.028), and more elevated preoperative CA125 levels (P = 0.014). Our study showed that there was no significant difference in uterine volume and CA125 levels in patients with early-stage adenomyosis with or without endometriosis (Table 1). Patients with early-stage extrinsic adenomyosis may already have endometriosis, despite small lesions and uterine volume. Furthermore. the B1 phenotype appears to correspond to the focal adenomyosis of the outer myometrium (FAOM) reported by Marcellin et al. [24]. The prevalence of FAOM was 56.5% in 255 women with DIE. Our results that endometriosis coexisted in 51.2% of the B1 group are similar to their report. FAOM and type B1 can develop from endometriosis, especially deeper invasive endometriosis. Khan et al. [25] also reported that the detection rate of coexisting DIE was significantly higher in women with extrinsic adenomyosis (9/10 [90.0%]) than in women with intrinsic adenomyosis (3/23 [13.0%]; P < 0.001). The difference from their study is that we focused on a large number of early-stage adenomyosis cases and examined the detection rate of not only coexisting DIE but also coexisting OMA or SUP.

Second, one of the key findings of our study, through univariate analysis, is that the previous history of induced abortion and curettage was identified as a risk factor for early-stage intrinsic adenomyosis. However, there were no significant differences between the A1 and B1 groups in the number of clinical pregnancy, live birth, and caesarean section (P = 0.070, 0.666, 0.727, respectively). Second, Increased childbirth, increased irregular menstrual cycles, a previous history of induced abortion and curettage, and smokers have already been reported as potential risk factors for adenomyosis [26]. Leyendecker et al. proposed that adenomyosis is caused by micro-trauma such as tissue injury and repair at the endometrial-myometrial interface [27]. These data suggest that endometrial trauma and inflammation might be closely involved in the development of intrinsic adenomyosis. The intrinsic type is a classic form of adenomyosis caused by the direct invagination of the basal endometrium into the myometrium, as previously thought [28]. This idea can be supported by our results focusing on early-stage intrinsic adenomyosis.

Third, there were no statistically significant differences in the frequencies of pelvic pain, menorrhagia, and infertility between the A1 and B1 groups. Pelvic pain was expected to be more frequent in group B1 patients due to the higher incidence of endometriosis, but results may be subject to selection bias because patients in group A1 with mild clinical symptoms do not undergo surgery. Adenomyosis has been thought to occur in multiparous women in the late reproductive age and present with pain and menorrhagia [1]. However, adenomyosis has come to be identified in symptomatic or asymptomatic nulligravid women earlier in reproductive life (early 20 years) by using modern imaging techniques such as transvaginal ultrasound and MRI [1,7]. It cannot be concluded that intrinsic adenomyosis is more common at older reproductive age, as there is no significant difference in age at surgery between groups A1 and B1. This suggests that onset and progression may be the same in both groups. However, this study could not determine the age at which the disease developed. In addition, no significant difference was found in preoperative hormonal therapies, including GnRH agonists, low-dose estrogen-progestin combinations (combined oral contraceptives), and dienogest, between the two groups. Clinicians do not seem to change hormone therapy depending on the type and size of adenomyosis.

Finally, the advantage of this study is that a rich and complete medical database was used to analyze the relationship between early-stage adenomyosis phenotype and clinicopathological characteristics. The disadvantage is that the lack of an internationally unified adenomyosis classification makes it difficult to compare our data with data from other studies. Moreover, patients who participated in this study were those who ultimately decided to undergo surgery due to a variety of subjective symptoms and are composed of a heterogeneous population.

In conclusion, early lesions of adenomyosis consist of at least two types with different etiologies. Patients with intrinsic adenomyosis are more likely to present with a history of induced abortion and curettage, and less likely to present with endometriosis, suggesting that mechanical damage of the endometrium may participate in this type of disease. Patients with intrinsic adenomyosis are more likely to present with a history of induced abortion and curettage, supporting the hypothesis that mechanical damage of the endometrium may participate in this type of disease. Extrinsic adenomyosis is often associated with endometriosis, even in the early stages. Further research is needed to provide evidence regarding the causal pathological relationship between extrinsic adenomyosis and endometriosis. A prospective study of patients with early lesions of adenomyosis diagnosed by MRI will allow the elucidation of the pathogenesis and disease heterogeneity.

## Conclusions

There are at least two types of adenomyosis, where the intrinsic type is closely associated with a history of induced abortion, while the extrinsic type is strongly associated with endometriosis.

## Supporting information

S1 File Adenomyosis data in English. (XLSX)
